# Functional Gene Expression in Shark Bay Hypersaline Microbial Mats: Adaptive Responses

**DOI:** 10.3389/fmicb.2020.560336

**Published:** 2020-11-16

**Authors:** Matthew A. Campbell, Kliti Grice, Pieter T. Visscher, Therese Morris, Hon Lun Wong, Richard Allen White, Brendan P. Burns, Marco J. L. Coolen

**Affiliations:** ^1^WA-Organic Isotope Geochemistry Centre, The Institute for Geoscience Research, School of Earth and Planetary Sciences, Curtin University, Perth, WA, Australia; ^2^Departments of Marine Sciences and Geoscience, University of Connecticut, Storrs, CT, United States; ^3^Australian Centre for Astrobiology, University of New South Wales, Sydney, NSW, Australia; ^4^Applied Geology, Curtin University, Perth, WA, Australia; ^5^School of Biotechnology and Biomolecular Sciences, The University of New South Wales, Sydney, NSW, Australia; ^6^Plant Pathology, Washington State University, Pullman, WA, United States; ^7^RAW Molecular Systems (RMS) LLC, Spokane, WA, United States

**Keywords:** hypersaline, metatranscriptomics, adaptive responses, microbial mat communities, Shark Bay (Australia)

## Abstract

Microbial mat communities possess extensive taxonomic and functional diversity, which drive high metabolic rates and rapid cycling of major elements. Modern microbial mats occurring in hypersaline environments are considered as analogs to extinct geobiological formations dating back to ∼ 3.5 Gyr ago. Despite efforts to understand the diversity and metabolic potential of hypersaline microbial mats in Shark Bay, Western Australia, there has yet to be molecular analyses at the transcriptional level in these microbial communities. In this study, we generated metatranscriptomes for the first time from actively growing mats comparing the type of mat, as well as the influence of diel and seasonal cycles. We observed that the overall gene transcription is strongly influenced by microbial community structure and seasonality. The most transcribed genes were associated with tackling the low nutrient conditions by the uptake of fatty acids, phosphorus, iron, and nickel from the environment as well as with protective mechanisms against elevated salinity conditions and to prevent build-up of ammonium produced by nitrate reducing microorganisms. A range of pathways involved in carbon, nitrogen, and sulfur cycles were identified in mat metatranscriptomes, with anoxygenic photosynthesis and chemoautotrophy using the Arnon–Buchanan cycle inferred as major pathways involved in the carbon cycle. Furthermore, enrichment of active anaerobic pathways (e.g., sulfate reduction, methanogenesis, Wood–Ljungdahl) in smooth mats corroborates previous metagenomic studies and further advocates the potential of these communities as modern analogs of ancient microbialites.

## Introduction

Microbial mats represent complex communities of microorganisms bound within an organic matrix of extracellular polymeric substances (EPSs) along with sediment and minerals (Des Marais, 1995; Wong et al., 2016). Their combined metabolic activities result in steep geochemical gradients that allows for the efficient cycling of nutrients (van Gemerden, 1993; Wimpenny et al., 2000). Microbial mats are found in a diverse range of environments around the world, including hypersaline lagoons (Dupraz and Visscher, 2005), geothermal hot springs (Kato et al., 2004), tidal flats (Jahnert and Collins, 2013), freshwater lakes (White et al., 2016), remnant asbestos mines (White et al., 2015), and freshwater karstic wetlands (Proemse et al., 2017). Microbial mats have persisted for ∼85% of the geological history of the Earth and have had major impacts on past global biogeochemical cycles, particularly oxygen, nitrogen, hydrogen, and sulfur (Visscher and Stolz, 2005). Modern microbial mats are considered as analogs to extinct geobiological formations with the oldest known fossils found in Western Australia, dating back ∼ 3.5 Gyr ago (Allwood et al., 2006; Van Kranendonk et al., 2008; Noffke et al., 2013).

Shark Bay in Western Australia has currently some of the most extensive extant microbial mat systems in the world occurring in meta- and hypersaline waters where they cover wide areas of intertidal and subtidal zones along ∼100 km of shoreline (Jahnert et al., 2012). Daily tidal cycles and meteorologically driven water level changes cause fluctuations in salinity, pH, temperature, desiccation stress, and UV radiation at a local scale adding to the extreme conditions present (Wong et al., 2018). Several different types of microbial mats have been recognized to occur in Shark Bay due to their surface morphology, namely gelatinous, smooth, tufted, pustular, and blister mats (Logan et al., 1972). These microbial mats present opportunities to investigate biological and physicochemical factors that influence their composition, morphology, and functionality (Allen et al., 2009; Suosaari et al., 2016). Previous taxonomic studies of Shark Bay microbial communities (e.g., 16S rRNA) have indicated a predominance of Proteobacteria, Bacteroidetes, Cyanobacteria, Chloroflexi, and Euryarchaeota (Burns et al., 2004; Goh et al., 2009; Wong et al., 2015). Furthermore, Shark Bay has high archaea diversity dominated by Parvarchaeota with a methanogenic community containing hydrogenotrophic Methanomicrobiales, as well as methylotrophic Methanosarcinales, Methanococcales, Methanobacteriales, and Methanomassiliicoccaceae (Wong et al., 2017). More recently, studies have examined the metagenomes of Shark Bay microbial mats. In these studies, the taxa and potential pathways associated with carbon, nitrogen, sulfur, and phosphorus cycles have been delineated, as well as environmental adaptations (e.g., low nutrients, hypersalinity, oxidative stress, and heavy metal resistance) (Ruvindy et al., 2016; Wong et al., 2018). However, it is currently unknown to what extent these pathways are actively transcribed in these extreme environments.

Metatranscriptomic profiling of microbial mats provides an opportunity to define key active metabolic pathways and adaptation mechanisms under different environmental conditions, as well as comparing active microbial populations from rRNA and mRNA transcripts. In Shark Bay microbial mat communities, the degree of which certain metabolic pathways (e.g., carbon fixation, oxygenic/anoxygenic photosynthesis, sulfate reduction) contribute to overall biogeochemical cycling and taxa responsible is still not fully understood. Here, we analyzed metatranscriptomes in Shark Bay microbial mats to investigate how active microbial communities, abundant gene transcription, and photosynthetic and chemosynthetic capacities change in accordance to mat type, diel cycle, and sampling season.

## Materials and Methods

### Sampling and Site Description

**FIGURE 1:**
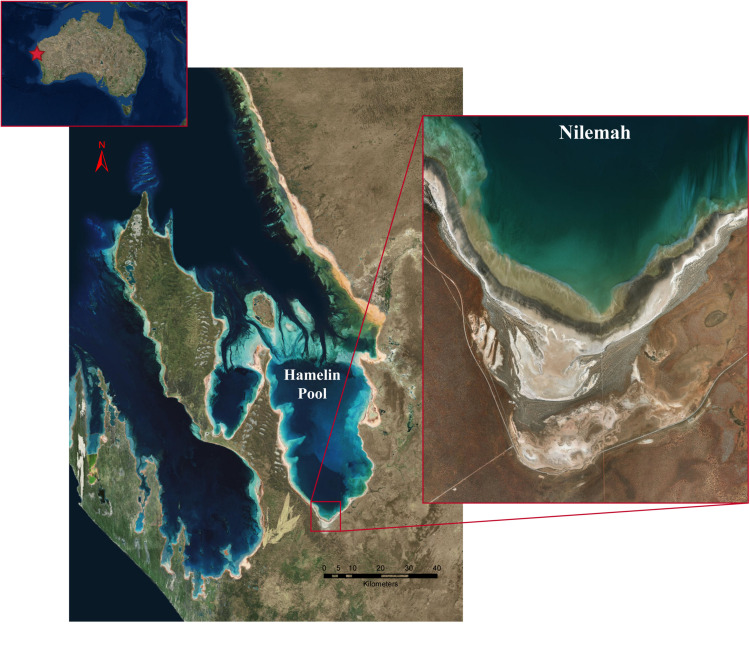
Satellite images of the Shark Bay world heritage area, Hamelin Pool, and the Nilemah tidal flat. Images were generated with ArcGIS Desktop: Release 10. Redlands, CA: Environmental Systems Research Institute.

**FIGURE 2:**
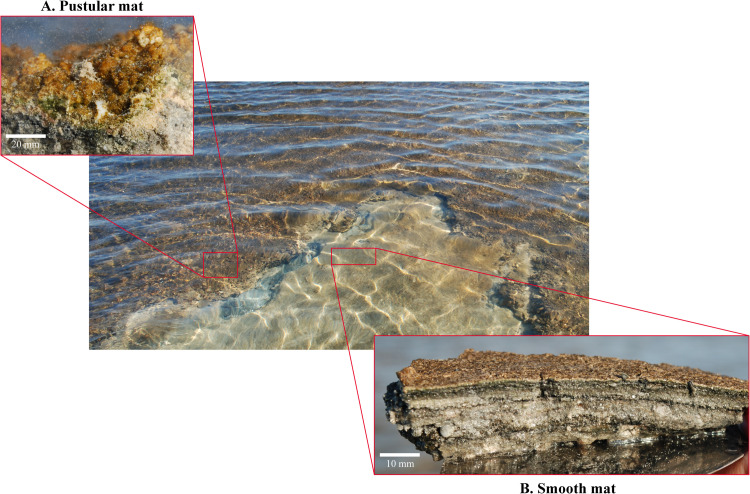
Smooth and pustular microbial mats sampled from the Nilemah tidal flat. **(A)** Pustular mat (bar, 20 mm) and **(B)** smooth mat (bar, 10 mm) collected in April 2017 from Nilemah.

Microbial mats were sampled from the Nilemah tidal flat located in the southern area of Hamelin Pool, Shark Bay, Western Australia (Figure 1). Restricted circulation in conjunction with high rates of evaporation and limited rainfall leads to the water in Hamelin Pool to be hypersaline (Hagan and Logan, 1974). Smooth and pustular microbial mats were sampled from the intertidal and uppermost subtidal zone of the Nilemah tidal flat (Figure 2). Smooth mats were uniformly laminated with a pale brown-green surface overlaying a light to dark green second layer followed by a third purple layer and fourth black layer and were located in small depressions that remained submerged at low tide between mat covered microbialite ridges. In contrast, pustular mats were dark brown at the surface with jelly-like pustules composed of green, gold, and purple mucilage. This mat type was located at on the crests of microbialite ridges between depressions in the shallow subtidal zone and as continuous sheets across the intertidal platform where it was exposed at low tide. These mat types were chosen for this study since they are the most common mat types for this area and parallel metagenomics datasets are available for comparison (Wong et al., 2018). In total, 10 microbial mats were sampled in the field using aluminum push cores for genomic analyses during the day and night at low tide periods in July 2016 and April 2017. Due to the rapidly changing tide over the Nilemah tidal flat, once the push cores were taken in the field, a subsection (∼5 cm^2^) of the top 10–30 mm of the “active” mat was immediately placed in a sterilized opaque plastic container with RNAlater (Thermo Fischer Scientific, MA, United States). The subsectioned mat samples were taken back to the field laboratory to be subsampled in triplicate with UV sterilized open-ended single-use 5 mL syringes and placed into sterile tubes containing RNAlater. Mats that were sampled during the daylight hours remained exposed to reduced sunlight at all times between field sampling and subsampling. After initial refrigerated storage for 24 h to allow the RNAlater to fully penetrate the samples, the tubes were stored and transported at −20°C and then stored at −80°C until nucleic acid extraction. In total, 10 microbial mats were collected from Nilemah over two field trips to Shark Bay in July 2016 and April 2017. See Supplementary Material for sampling dates and times, and field measurements (Supplementary Table 1), as well as more detailed site and sample descriptions.

### RNA Isolation, Library Preparation, and Sequencing

The RNeasy PowerSoil Total RNA Kit (Qiagen, Hilden, Germany), which subjects samples to efficient chemical and mechanical (bead beating) cell lysis, was used to extract total RNA (in triplicate) from 50 to 100 mg of sample. An additional DNAse treatment with the Turbo DNA-free Kit (Qiagen), was used to remove residual DNA and the DNA-free RNA extracts were purified using the MEGAclear kit (Thermo Fischer Scientific). RNA concentrations were measured with a NanoDrop 3300 (Thermo Fisher Scientific), using the Quant-iT^TM^ RiboGreen^TM^ RNA Assay Kit (Thermo Fisher Scientific), and a SYBR green based quantitative PCR targeted bacterial 16S rDNA (V4 region) was used to test if the DNase-treated RNA samples were completely free of DNA. Equimolar amounts of the triplicate DNA-free RNA samples were pooled for the synthesis of cDNA using the Ovation RNAseq System V2 kit and subsequent library preparation using the Ovation Ultralow Library System V2 kit (NuGEN technologies, CA, United States) following manufacturers protocol. cDNA libraries were analyzed for quality and quantity with a 2100 bioanalyzer (Agilent, CA, United States) and subsequently sent to the Australian Genomic Research Facility (AGRF) for sequencing. At AGRF, the Illumina HiSeq 2500 platform was used to generate 2 × 125-bp pair-end sequencing reads. The HiSeq Control Software (HCS) v2.2.68 and Real-Time Analysis (RTA) v1.18.66.3 software performed real-time image analysis and base calling on the HiSeq instrument computer. The AGRF Illumina bcl2fastq 2.20.0.422 pipeline was used to generate the sequence data.

### Metatranscriptomic Analysis

Taxonomic analysis of the microbiota was based on assembled 16S rRNA transcripts, followed by functional and taxonomic annotation of transcripts derived from the mRNA fraction. Metatranscriptomic analysis was achieved by trimming raw paired-end Illumina sequenced reads with BBDuk; BBDuk was ran twice, first to trim adapters with parameters ktrim = *r*, *k* = 21, mink = 11, hdist = 2, tpe, tbo and then again for quality trimming with parameters qtrim = *r*, trimq = 25, maq = 25, minlen = 50, *k* = 31, qhdist = 1 (Bushnell, 2018). Bowtie2 was used to map rRNA and phiX sequences then Samtools was used to separate and convert sequences into mapped and unmapped fastq files (Li et al., 2009; Langmead and Salzberg, 2012). Fastq-pair was used to match the pair-end mapped and unmapped reads to ensure that all reads were paired and to separate out singletons (Edwards and Edwards, 2019). For taxonomic analysis of the mapped reads (rRNA sequences), the pair-end mapped reads were analyzed using the Phyloflash pipeline (v.3.3). Reconstructed 16S and 18S rRNA gene sequences were aligned to sequences from the SILVA small subunit (SSU) 132 NR99 database with BBmap, with minimum identity of 85% and read limit of <5,000,000 bp (Gruber-Vodicka et al., 2019). For functional and taxonomic analysis of unmapped reads (mRNA sequences), the unmapped pair-end reads were checked again for presence of rRNA and phiX sequences using Bowtie2 then assembled into transcripts using rnaSPAdes (v.3.13.0) with the default *k*-mer size of *k* = 55 (Bushmanova et al., 2019). Protein sequences predicted with Prodigal (v.2.6.1) were then annotated using eggnog-mapper (v.1.0.3) in “diamond” run mode with the eggNOG database (v.4.5.1) for functional analysis (Hyatt et al., 2010; Huerta-Cepas et al., 2017). Kegg orthologs (KOs), enzymes, pathways, and modules were inferred from the eggnog-mapper output using the Kegg brite hierarchy information (Kanehisa et al., 2008). Taxonomic assignments were achieved by mapping the predicted proteins using DIAMOND blastp aligner with the default settings in more sensitive mode; reads were mapped against NCBI’s most recent RefSeq non-redundant protein release. Illumina HiSeq 2500 pair-end sequencing read output with percentage summaries of trimmed and aligned sequences, number of assembled transcripts and number of annotations for Phyloflash, DIAMOND and eggnog-mapper can be found in Supplementary Table 2.

### Data Analysis

Alpha diversity indices (Chao1 richness and Shannon diversity) of the SILVA SSU (rRNA) and RefSeq classifications (mRNA) were evaluated with the R library phyloseq (McMurdie and Holmes, 2013). Two-way analysis of variance (ANOVA) followed by Tukey’s *t*-test (*P*-value > 0.05) of the taxonomic distributions (phyla level, as well as class level for Proteobacteria) was performed using Statistical Analysis of Metagenomics Profiles (STAMP) (Parks et al., 2014). Principle component analysis (PCA) of normalized SILVA (SSU rRNA) and RefSeq classifications was performed with R libraries Ecodist (dissimilarity-based functions for ecological analysis), and pvclust (hierarchical clustering with *P*-values via Multiscale Bootstrap Resampling) using ward clustering and Bray–Curtis distance metrics at a thousand replicates. Dot plots of normalized SILVA (SSU rRNA) and RefSeq classifications were completed using R libraries Reshape2 with the melt function then plotted using ggplot2 (Wickham, 2011). ANOSIM (max of 999 permutations) using a one-way ANOVA model with Spearman Rank correlation was performed on normalized and square root transformed functional gene transcript data at single function level in PRIMER-e v7 to reveal if microbial functions differed significantly (*P* < 0.05) between mat types, diel cycles, and sampling period (Clarke and Gorley, 2006). Heatmaps of transcribed genes distributions were prepared by differential analysis calculated from the variance stabilizing transformation of KO count data using the DESeq2 package and visualization using pheatmap in R. The STAMP Welch’s *t*-test was completed against mat type and day/night samples using the one-sided, CI method with no multiple test correction using Benjamini–Hochberg FDR to find significant differences between metabolic pathways. Transcribed gene counts lower than 10 were excluded from the above analyses. Additionally, samples were compared to a procedural blank to evaluate if any discrepancies in the data was caused by contamination.

## Results

### Microbial Community Structure of Smooth and Pustular Mats

The alpha diversity of microbial community structure between smooth and pustular mats was estimated for both rRNA and mRNA derived classifications with the Shannon diversity index and Choa1 species richness estimator. Shannon index of diversity for rRNA classifications ranged from 5.66 to 6.12 in pustular mats, averaging higher than smooth mats that ranged from 5.25 to 5.75 (Supplementary Figure 1A). Interestingly, the mRNA showed an opposite trend, ranging from 6.10 to 6.29 in smooth mats, averaging higher than pustular mats that ranged from 5.11 to 6.10 (Supplementary Figure 1B). The Chao1 species richness estimator revealed that richness for rRNA classifications varied from 2260 to 3800 in pustular mats and from 2020 to 3250 in smooth mats with the highest values found in pustular mats sampled in April 2017 (Supplementary Figure 1A). Coincidently, mRNA classifications revealed that smooth mats had an overall higher species richness varying from 1580 to 1800, whereas pustular mats varied from 1290 to 1690 (Supplementary Figure 1B).

**FIGURE 3:**
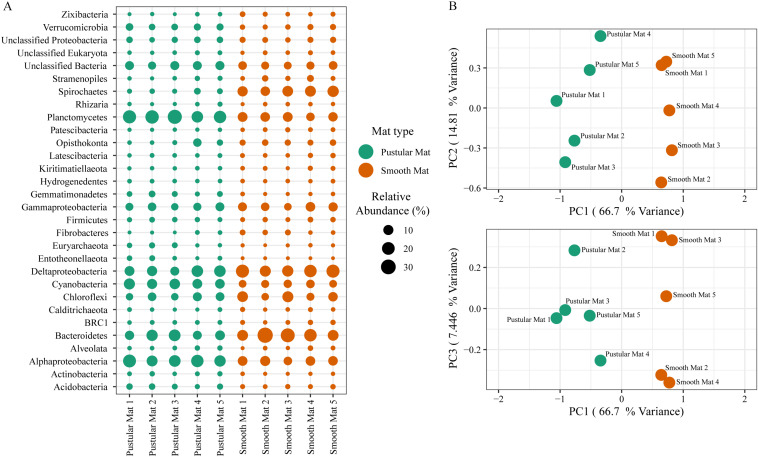
Composition of Archaea, Bacteria, and Eukaryote taxa in Nilemah smooth and pustular mats based on SSU rRNA genes (SILVA Database). **(A)** Dot plot displaying the composition and abundance of the top 30 most abundant taxa. **(B)** PCA plots constructed from similarity matrices. Mats 1, 2, and 3 were sampled in July 2016; mats 4 and 5 were sampled in April 2017. Pustular and smooth mats 2 and 3 are the paired samples from July 2016.

**FIGURE 4:**
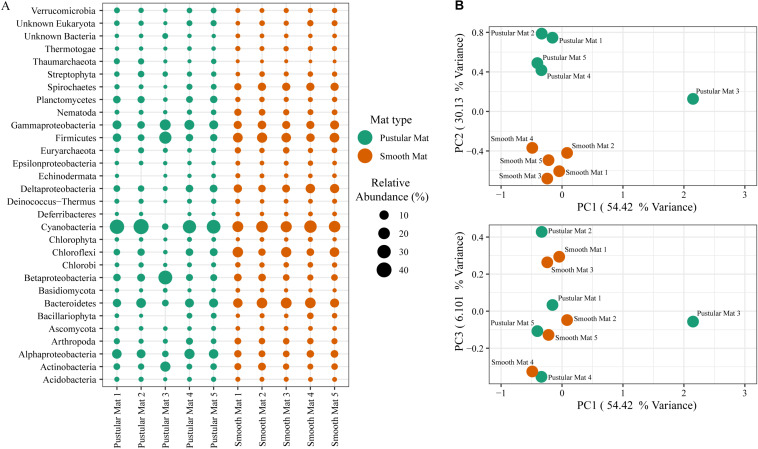
Composition of Bacteria, Archaea, and Eukaryote taxa in Nilemah smooth and pustular mats based on transcripts (RefSeq Database). **(A)** Dot plot displaying the composition and abundance of the top 30 most abundant taxa. **(B)** PCA plots constructed from similarity matrices. Mats 1, 2, and 3 were sampled in July 2016; mats 4 and 5 were sampled in April 2017. Pustular and smooth mats 2 and 3 are the paired samples from July 2016.

Principle component analysis of rRNA and mRNA classifications revealed that smooth mats are distinct from pustular mats, however the mRNA classification indicated a significant discrepancy with pustular mats sampled in 2016 during the night (Figures 3B, 4B). Based on both classification methods the community composition was primarily dominated by bacteria with a general proportion of 90:3:7% of Bacteria, Archaea, and Eukaryotes, respectively. Smooth and pustular mats were dominated by Proteobacteria, predominately the alpha, delta and gamma classes, as well as Bacteroidetes, Planctomycetes, Cyanobacteria, Spirochaetes, Firmicutes, and Chloroflexi (Figures 3A, 4A). Furthermore, mRNA classifications indicated that Betaproteobacteriales were also abundant but are not shown in the rRNA classifications, since this former bacterial class has been included within Gammaproteobacteria as Betaproteobacteriales in the updated Silva 132 database. Lower abundant taxa (>1%) can be seen in Supplementary Figures 2A, 3A.

The rRNA classifications indicated that Cyanobacteria, Planctomycetes, Verrucomicrobia, Amoebozoa, Alphaproteo- bacteria, Acidobacteria, Gemmatimonadetes, Thaumarchaeota, Nitrospirae, Omnitrophicaeota, and Actinobacteria were significantly more represented in the pustular mats (*P*-value < 0.05), whereas, smooth mats contained significant proportions of Spirochaetes, Patescibacteria, Deltaproteo- bacteria, Dependentiae, Modulibacteria, Asgardeota, and Nanoarchaeota (Supplementary Figure 2B). The mRNA classifications indicated that Planctomycetes and Alphaproteobacteria were significantly higher in pustular mats (*P*-value < 0.05), whereas Spirochaetes, Thermotogae, Deferribacteres, Chrysiogenetes, Synergistetes, Aquificae, Chlorobi, Deltaproteobacteria, Dictyoglomi, Bacteroidetes, Fusobacteria, Choroflexi, and Epsilonproteobacteria were more abundant in smooth mats (Supplementary Figure 3B). Both classifications indicated that Alphaproteobacteria and Planctomycetes were significantly more represented in pustular mats, whereas Deltaproteobacteria and Spirochaetes were more significant in smooth mats.

### Key Functional Gene Distribution in Metatranscriptomes

**FIGURE 5:**
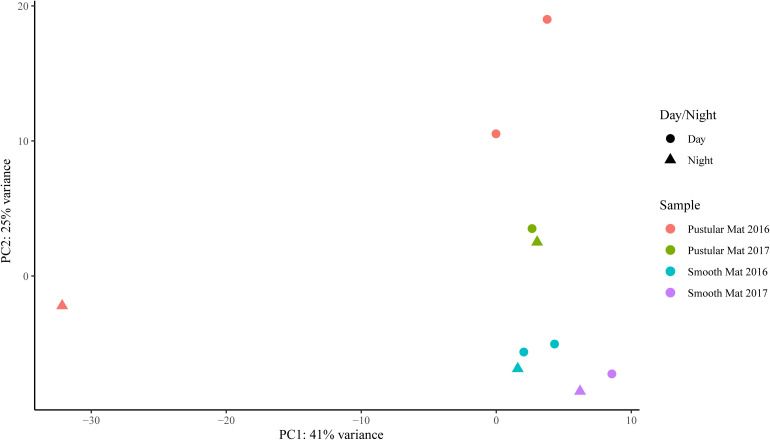
Principle component analysis showing relationships between mat types, day/night samples and sampling period. Differential analysis of the transcribed genes was calculated from the variance stabilizing transformation of KO count data using the DESeq2 package in R.

**FIGURE 6:**
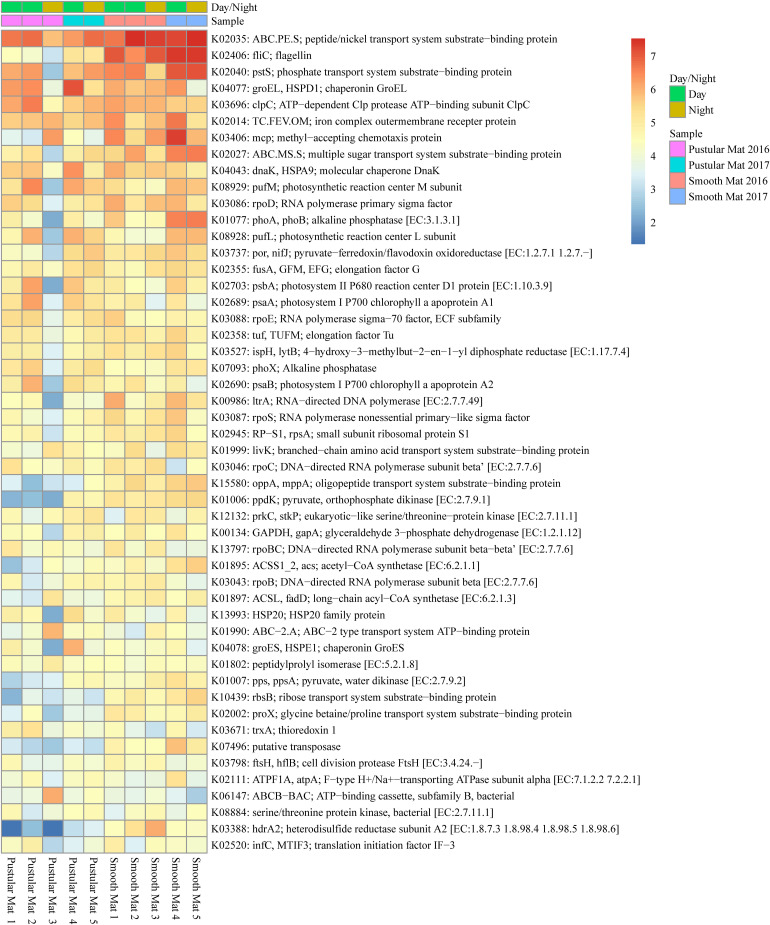
Heatmap showing the top 50 out of 2298 transcribed genes (>10) in Nilemah smooth and pustular mat metatranscriptomes. Differential analysis of the transcribed genes was calculated from the variance stabilizing transformation of KO count data using the DESeq2 package in R. A gradient from red to blue indicates gene abundance across samples with red representing genes that are highly transcribed and blue indicating genes that have lower relative transcription. Pustular and smooth mats 2 and 3 are the paired samples from July 2016.

Transcripts of genes for microbial functions only differed significantly between smooth and pustular mats (ANOSIM, *R* = 0.34; *P* = 0.008), but not between diel cycles and sampling period (*P* > 0.5). PCA of all transcribed genes indicated that there was no clustering observed between day and night samples. However, smooth mats clustered significantly more than pustular mats with the majority of the samples clustering within their sampling period (Figure 5). A large portion of the most differentially expressed transcripts (fold change >4) were associated with maintenance of basic cellular functions (e.g., chaperones, elongation factors, RNA polymerase) for both mat types. However, high levels of gene transcripts that encode quorum sensing (ABC.PE.S; ABC.MS.S; *opp*A, *mpp*A; *liv*K; ABC-2.A), motility/chemotaxis (*fli*C, *mcp*, *rbs*B), environmental adaptation (*clp*C, *pro*X), biofilm formation (*rpoS*), phosphorus uptake (*pst*S; *pho*ABX), iron uptake (TC.FEV.OM), terpene biosynthesis (*isp*H, *lyt*B) and fatty acid metabolism (ACSL, *fad*D) were observed (Figure 6). Furthermore, genes involved in photosynthesis (*puf*LM; *psa*A, *psb*A), as well as carbon metabolisms relating to carbon fixation and methane metabolism (*por*, *nif*J; *ppd*K; GAPDH, *gap*A; *hdr*A2; ACSS1_2; *pps*, *pps*A) were also highly abundant in both mat types. Additionally, both the PCA and heatmap (Figures 5, 6) confirmed the same discrepancy observed in mRNA classification of the 2016 pustular mats sampled during the night.

### Key Functional Gene Distribution in Microbial Metabolic Pathways

#### Photosynthesis

An abundance of transcripts encoding photosystems I (*psa*ABCDEFL) and II (*psb*ABCDEFOTUV) were found in both mat types, however the most differentially transcribed phototrophy-related genes (fold change >5) were associated with anoxygenic photosystem II (PSII; *puf*BLM) (Supplementary Figure 4). Other abundantly transcripts relating to phototrophy included the cytochrome b6/f complex (*pet*ABCD), F_1_F_0_-type ATPase (*atp*ABCDEFGH), and photosynthetic electron transport (*pet*CEH).

#### Carbon Fixation

The reductive citric acid cycle (Arnon–Buchanan cycle) had the highest abundance and proportion of transcripts related to carbon fixation across all metatranscriptomes (*por*, *nif*J; *ppd*K; *pps*, *pps*A; *por*A; *kor*ABC, *oor*ABC, *ofor*ABC; *sdh*ABCD, *frd*ABCD; ACO, *acn*A; *mdh*; IDH1, IDH2, *icd*; *suc*C; IDH1, IDH2, *icd*; *fum*C, FH; *suc*D; *por*B; *pyc*B; PC, *pyc*) (Supplementary Figure 5). The reductive pentose phosphate cycle (Calvin–Benson cycle) had the second highest abundance and proportion of transcripts (GAPDH, *gap*A; *tkt*A, *tkt*B; *rbc*L; FBA, *fba*A; PGK, *pgk*; PRK, *prk*B; *gap*2; *glp*X; *glp*X-SEBP; *rbc*S; FBP, *fbp*; ALDO). Genes involved in reductive acetyl-CoA pathway (Wood–Ljungdahl pathway) were abundantly transcribed in the smooth mat metatranscriptomes (*fhs*; *coo*S, *acs*A; *fol*D; *acs*BC; *fdh*AB; *met*F, MTHFR; *cdh*E). Transcripts of genes involved in 3-hydroxypropionate bi-cycle (*sdh*ABC, *frd*ABC; MUT; *mcm*A1; *fum*C, FH; *acc*ACD), as well as the hydroxypropionate-hydroxybutyrate cycle (*ato*B, *mcm*A1) were transcribed in both mat types.

#### Methane Metabolism

**FIGURE 7:**
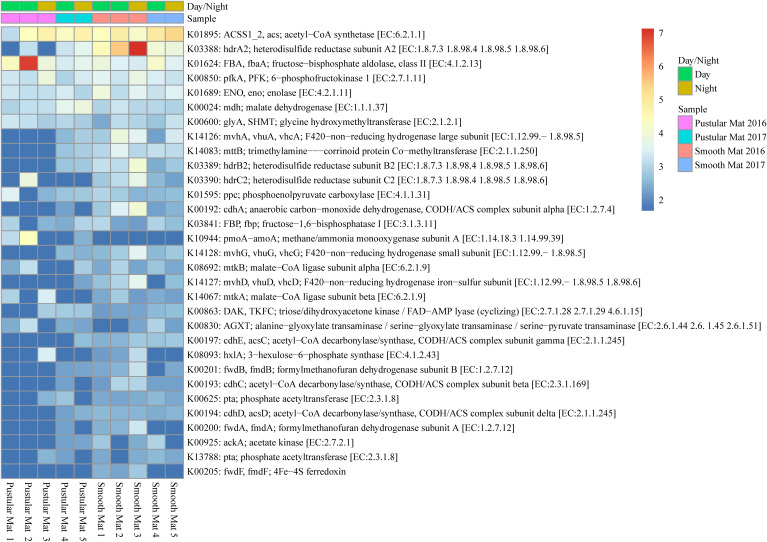
Heatmap showing 31 transcribed genes (>10) in Nilemah smooth and pustular mat metatranscriptomes related to methane metabolism. Differential analysis, gradient, and sample descriptions are the same as Figure 4.

Genes involved in methane metabolism were found to be abundantly transcribed (fold change >4) within smooth mat metatranscriptomes (Figure 7), with the most abundant transcripts associated with the final reaction steps of the methanogenic pathway (*hdr*ABC2; *mvh*ADG, *vhu*ADG, *vhc*ADG). Two major types of methanogenic pathways were found to be transcribed, CO_2_ to methane (*fwd*ABF, *fmd*ABF) and acetate to methane (ACSS1_2, *acs*; *pta*; *ack*A; *cdh*E, *acs*C; *cdh*C; *cdh*D, *acs*D), as well as the less common trimethylamine to methane (*mtt*B). Moreover, transcription of genes involved in pathways that convert formaldehyde to C2 or C3 compounds were also highly transcribed between mat types, these include the serine pathway (ENO, *eno*; *mdh*; *gly*A, SHMT; *ppc*; *mtk*AB; AGXT) and ribulose monophosphate pathway (FBA, *fba*A; *pfk*A, PFK; *hxl*A). Interestingly, methanotrophic oxidation of methane to formaldehyde was only observed in pustular mat metatranscriptomes (*pmo*A-*amo*A), however, this gene is also associated with encoding nitrification. Furthermore, transcription of genes associated with the acetyl-CoA pathway (Wood–Ljungdahl pathway) were also observed (*cdh*ACDE; *acs*CD).

#### Nitrogen Metabolism

Transcription of genes that encode nitrogen metabolizing pathways showed no clear pattern between mat types (Supplementary Figure 6). Transcription of genes involved with the breakdown and biosynthesis of glutamate were heavily represented in all metatranscriptomes (*gdh*A; *glt*BD; GLU, *glt*S; GLUD1_2, *gdh*A; GLT1; *gud*B, *roc*G; GDH2), with glutamine synthetase (*gln*A, GLUL) being the most abundantly transcribed nitrogen metabolism related gene. Genes involved in nitrogen fixation were also abundantly transcribed (*nif*DHK) in both mat types, however they were found to be more abundantly transcribed in samples collected during the night in April 2017. Other reduction pathways found in the mats included assimilatory nitrate reduction (*nar*B, *nir*A, *nas*A), dissimilatory nitrate reduction (*nar*G, *nar*Z, *nxr*A; *nap*A; *nrf*A), and denitrification (*nar*G, *nar*Z, *nxr*A; *nos*Z; *nap*A; *nir*K). Additionally, genes encoding ammonia/bicarbonate cycling (*cyn*T, *can*; *arc*C; CPS1), hydroxylamine reduction to ammonia (*hcp*), nitroalkane assimilation (*ncd*2, *npd*), nitrate assimilation (NRT, *nark*, *nrt*P, *nas*A), nitrile assimilation (E3.5.5.1), and formamide assimilation (E3.5.1.49) were transcribed across all samples studied.

#### Sulfur Metabolism

**FIGURE 8:**
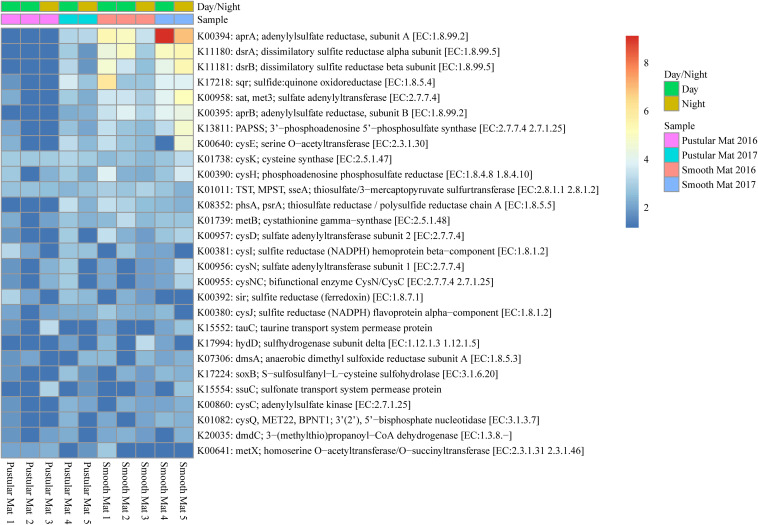
Heatmap showing 28 transcribed genes (>10) in Nilemah smooth and pustular mat metatranscriptomes related to sulfur metabolism. Differential analysis, gradient, and sample descriptions are the same as Figure 4.

Genes involved in sulfur metabolism were found to be abundantly transcribed (fold change >4) within the smooth mat metatranscriptomes (Figure 8), with the most abundant genes being associated with the dissimilatory sulfate reduction pathway (*apr*AB; *dsr*AB; *sat*, *met*3) and assimilatory sulfate reduction pathway (*sat*, *met*3; *cysCD*HIJNQ; PAPSS; *sir*). Genes encoding polysulfide metabolism (*sqr* and *hyd*D), thiosulfate reduction (TST, MPST, *sse*A; *phs*A, *psr*A), cysteine and methionine metabolism (*cys*K, *cys*E, *met*B, *met*X), dimethyl sulfoxide reduction (*dms*A), taurine assimilation (*tau*C), thiosulfate oxidation (*sox*B), alkanesulfonate assimilation (*ssu*C), and assimilation of dimethylsulphoniopropionate (*dmd*C) were transcribed across all samples studied.

### Significant Metabolic Activity

**FIGURE 9:**
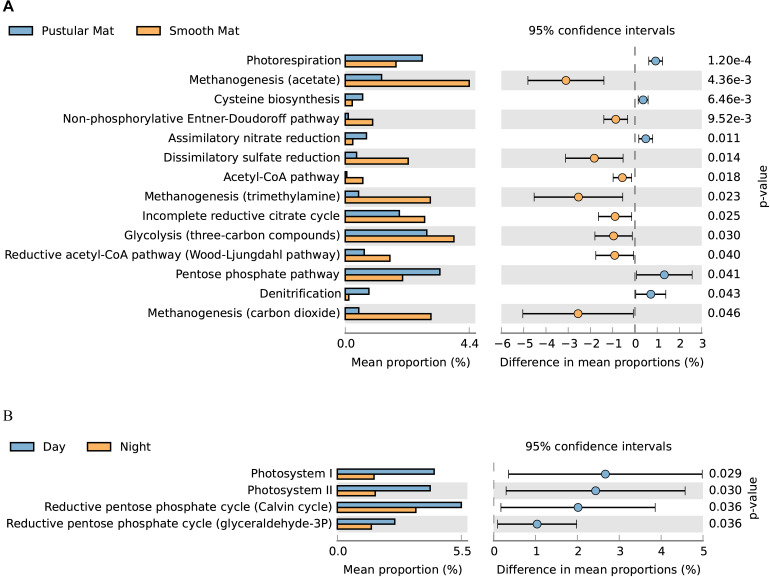
Extended error bar plot identifying significant differences (*P* < 0.05) between mean proportions of 56 metabolic pathways (KEGG level 3) in smooth and pustular mats **(A)**, and day and night samples **(B)** (Welch’s *t*-test).

Overall expression of metabolic pathways between mat types indicated that photorespiration, cysteine biosynthesis, nitrate assimilation, denitrification, and the pentose phosphate pathway were significantly represented in pustular mats, whereas the metabolic activity in smooth mats was more represented by anaerobic pathways that included methanogenesis, non-phosphorylative Enter–Doudoroff pathway, acetyl-CoA pathway, dissimilatory sulfate reduction, and the Wood–Ljungdahl pathway (Figure 9A). Comparison of day and night samples indicated that transcription of genes involved in photosystems I and II, as well as the reductive pentose phosphate cycle (glyceraldehyde-3P) and reductive pentose phosphate cycle (Calvin cycle) were more abundant during the day time (Figure 9B).

## Discussion

### Active Microbial Mat Communities

The overall microbial community composition of Nilemah smooth and pustular mats exhibited similar distributions to previous metagenomic and 16S rRNA/rDNA studies of Nilemah microbial mats and stromatolites (Burns et al., 2004; Allen et al., 2009; Ruvindy et al., 2016; Wong et al., 2016; Babilonia et al., 2018). This suggests that the microbial communities in the Nilemah tidal flat have been relatively stable for the last two decades, dominated by Proteobacteria, Bacteroidetes, Planctomycetes, Cyanobacteria, Spirochaetes, Firmicutes, and Chloroflexi occupying the mats. Comparison of active microbial communities derived from rRNA and mRNA transcripts in both mat types indicated that pustular mats had an overall higher species richness and diversity than smooth mats based on rRNA classifications, however an opposite trend was observed with transcript derived mRNA classifications suggesting that the density of active microbes in smooth mats is relatively higher. This corroborates with depth profiles of oxygen and sulfide concentrations in Shark Bay microbial mats (Supplementary Figure 7), as well as previous studies (e.g., Wong et al., 2015), which indicated that smooth mats had higher cell densities due to the increased metabolic activity of aerobes and intense cycling of carbon in their upper layers when compared to pustular mats. PCA of both classifications revealed that the microbial community compositions of smooth mats are distinct from pustular mats, however the mRNA classification indicated a significant discrepancy with a 2016 pustular mat sampled during the night. This discrepancy is not observed in the rRNA classification, therefore indicating that this particular sample is showing a possible shift in the active microbial community with Gammaproteobacteria, Firmicutes, Betaproteobacteriales, and Actinobacteria becoming more active during the night, and Cyanobacteria, Bacteroides, and Alphaproteobacteria becoming less active.

### Microbial Mats Surviving in a Low Nutrient, Hypersaline Environment

A large portion of most abundantly transcribed genes were associated with maintaining basic cellular functions. However, the metatranscriptomes exhibited elevated expressions of genes associated with combating hypersalinity and low nutrient conditions such as biofilm formation, nutrient uptake, and osmoadaptation. Our study found an abundance of genes encoding sigma factor rpoS, ATP-binding subunit ClpC and transport systems associated with quorum sensing (QS), which are all linked with biofilm formation and are recognized as a key factors in the stationary phase of growth and survival of bacteria during exposure to stress conditions (i.e., starvation, UV radiation, salinity, and oxidative stress) (Muñoz-Elías et al., 2008; Klauck et al., 2018; Lee et al., 2018). Moreover, the most transcribed gene found across all metatranscriptomes encodes a peptide/nickel ABC transporter associated with transcriptional regulators that serve as key components of QS pathways which utilize peptides as intercellular signaling molecules aiding in virulence and biofilm formation (Rutherford and Bassler, 2012; Papenfort and Bassler, 2016). However in bacteria, peptide/nickel ABC transporters are also linked with nickel uptake, and as soluble Ni_2_^+^ in natural environments usually exists in trace amounts, high-affinity uptake of nickel is needed to ensure intracellular metalloenzyme activities (Hiron et al., 2010; Zhu et al., 2017). Previous analysis of the a Shark Bay smooth mats found the nickel concertation to be at 7 ppm, an adequate concentration for (NiFe) hydrogenase activity (Skyring et al., 1988). Additionally, sub-millimolar concentrations of nickel can inhibit biofilm formation in bacteria through the inhibition (at the transcriptional level) of QS (Vega et al., 2014). Therefore, high expression of this peptide/nickel ABC transporter suggests that microorganisms that utilize nickel in Shark Bay microbial mats need to highly regulate the intracellular concentrations of nickel to ensure adequate levels for metalloenzyme activities while not inhibiting QS.

Genes involved in the uptake and regulation of iron, phosphorus, and long chain fatty acids were also abundantly transcribed in the metatranscriptomes. A variety of nutrients are known to be limited in Shark Bay microbial mats (i.e., phosphorus), with their distribution fluctuating in accordance to oxic and anoxic conditions (Pagès et al., 2014; Wong et al., 2018). The high abundance of an inorganic phosphate transport gene (*pst*S) is likely critical for inorganic phosphorus uptake, with its accumulation being enhanced under phosphate starvation conditions (Varin et al., 2010). Genes encoding alkaline phosphatase (*pho*ABX) were also abundant throughout the metatranscriptomes. Alkaline phosphatase is responsible for salvaging phosphorus from organic molecules in the community (Chróst, 1994). A gene regulating the iron complex outer membrane receptor protein (TC.FEV.OM) was also highly transcribed; this protein regulates intracellular iron and prevents its over accumulation (Mackenzie et al., 2008). Another abundantly transcribed gene in the metatranscriptomes encodes long-chain acyl-CoA synthetase (ACSL, fadD) known for catalyzing the activation of a long fatty acid chain to a fatty acyl CoA, and has key involvement in obtaining essential fatty acids from the environment to then be oxidized for energy production (Dirusso and Black, 2004).

Genes enhancing a microbe’s ability to sense chemical gradients in their environment and then move toward more favorable conditions were also abundantly transcribed in all samples studied, but were especially transcribed in smooth mat metatranscriptomes. These include the flagellin protein (*fli*C), the methyl-accepting chemotaxis protein (*mcp*) and the ribose transport system substrate-binding protein (*rbs*B). Methyl-accepting chemotaxis proteins are the most common receptors in Bacteria and Archaea (Salah Ud-Din and Roujeinikova, 2017). The *rbs*B protein is associated with the methyl-accepting chemotaxis protein III, which is influenced by D-ribose, D-galactose, and certain structural analogs (Kondoh et al., 1979). The overexpression of the *fli*C gene has been associated with the enhancement of motile activity; however, deletion of the *fli*C gene has also been found to cause an inhibited in QS activity and biofilm formation. This suggests that the *fli*C gene may have a variety of functional properties (Morohoshi et al., 2009; Zhou et al., 2014). The higher abundance of these genes in the smooth mats is likely a result of the heterotrophic bacteria needing to adjust their position within these stratified systems during diel fluctuations that change the oxic and anoxic conditions. Furthermore, a previous study found evidence of an anoxic niche occurring in the upper photic-oxic zone of Shark Bay microbial mats with large portions of anaerobic bacteria, such as Spirochaetes, occurring in the oxygenated surface layer (Wong et al., 2015).

Previous functional annotations of Shark Bay metagenomes have identified clusters of osmoadaptive traits such as the uptake of the osmoprotectant glycine betaine (Wong et al., 2018). In this study genes encoding glutamine synthetase (*gnl*A, GLUL) and glutamate synthase (*glt*D) were highly represented in all metatranscriptomes. Glutamate is known to be a major anionic solute involved in osmoregulation (Kang and Hwang, 2018). However, in most photosynthetic organisms, ammonium assimilation takes place by the sequential action of glutamine synthetase and glutamate synthase, constituting a link between nitrogen and carbon metabolisms (Muro-Pastor et al., 2005). Cycling of glutamate could be critical in the Shark Bay systems as a protective mechanism against elevated salinity conditions as well as potential source of carbon and energy in times of stress. Furthermore, genes transcribing F_1_F_0_-type ATP synthase relating to H^+^/Na^+^ transport were abundantly transcribed in both mat types. The F_1_F_0_-type ATP synthase plays vital functions in the energy-transducing membranes of bacteria by catalyzing ATP synthesis and hydrolysis coupled with transmembrane proton or sodium ion (Na^+^) transport (von Ballmoos et al., 2009). To survive at high salinity, organisms must prevent the excessive Na^+^ accumulation in the cytoplasm (Assaha et al., 2017). Studies on halotolerant cyanobacteria have indicated that they potentially utilize Na^+^-dependent F_1_F_0_-type synthase to tolerate salt-stress in conjunction with more common osmoprotectants such as glycine betaine (Soontharapirakkul et al., 2011). Therefore, the elevated expression of genes encoding H^+^/Na^+^ dependent F_1_F_0_-ATP synthase maybe a significant osmoadaptation for photosynthetic organisms living in the hypersaline mats of Shark Bay.

### Carbon Cycling

Photosynthesis is (one of) the primary driving force(s) of various nutrient cycles in microbial mats (van Gemerden, 1993; Mobberley et al., 2013). The most abundantly transcribed phototrophy-related genes found in both mat types were involved in anoxygenic PSII. Anoxygenic photosynthesis is distinguished from oxygenic photosynthesis by the terminal reductant (e.g., hydrogen sulfide) and in the by-product generated (e.g., zerovalent sulfur) (Visscher et al., 1992; van Gemerden, 1993; Soontharapirakkul et al., 2011). The abundant *puf*LM transcripts encode photosynthetic reaction center L and M proteins involved in the Type II reaction centers which are known occur in bacteria such as Chloroflexi, Proteobacteria, and Gemmatimonadetes (Masuda et al., 2000; Zeng et al., 2014). Cyanobacteria, when conducting oxygenic photosynthesis use PSII and a Type I reaction center known as photosystem I (PSI), which were also abundantly transcribed within the metatranscriptomes. The reductive pentose phosphate cycle (Calvin–Benson cycle) regarded quantitatively as the most important mechanism of autotrophic CO_2_ fixation in nature, is a feature of oxygenic photosynthesis in cyanobacteria, as well as anoxygenic photosynthesis in purple sulfur bacteria and chemolithotrophic (including sulfide/sulfur-oxidizing) bacteria (Lengeler et al., 1998; Berg, 2011). However, the Calvin–Benson cycle had the second highest level of transcription in contrast to the reductive citrate cycle (Arnon–Buchanan cycle), which is typically found in anoxygenic photosynthetic green sulfur bacteria (Chlorobi), as well as in various anaerobic or microaerobic members of Aquificae, Proteobacteria, and Nitrospirae (Hügler and Sievert, 2011). Interestingly, green sulfur bacteria use anoxygenic PSI and the Arnon–Buchanan cycle, but no genes were transcribed for anoxygenic PSI in any of the metatranscriptomes studied, suggesting that this carbon fixation pathway is primarily used by chemosynthetic organisms residing in Shark Bay microbial mats. The higher abundance of genes transcribing anoxygenic PSII could be ascribed to either Alphaproteobacteria that utilize the Calvin–Benson cycle or to the significant presence of green non-sulfur bacteria (Chloroflexi) that utilize the 3-hydroxypropionate bi-cycle to which was another abundantly transcribed carbon fixation pathway found in both mat types. On a further note, the Calvin–Benson cycle generally operates in situations of high light intensity, therefore the presence of this pathway is primarily associated with Cyanobacteria and Alphaproteobacteria occurring in the upper layers of the mats, whereas, under low light conditions, oligotrophic organisms use the more energetically favorable Arnon–Buchanan cycle (Berg, 2011).

Methanogenesis coupled to the reductive acetyl-CoA pathway (Wood–Ljungdahl pathway) is possibly one of the most ancient metabolisms for energy generation and carbon fixation (Borrel et al., 2016). Genes associated with the Wood–Ljungdahl pathway along with methanogenic pathways (i.e., CO_2_ to methane and acetate to methane) were over-represented in smooth mat metatranscriptomes. This corroborates with a previous study of Shark Bay smooth mats which reported higher rates of methane production in the presence of H_2_/CO_2_ as the methanogenic substrates (Wong et al., 2017). The Wood–Ljungdahl pathway is found strictly in anaerobic bacteria and archaea, such as Proteobacteria, Planctomycetes, Spirochaetes, and Euryarchaeota, that utilize a bifunctional enzyme, carbon monoxide dehydrogenase/acetyl-CoA synthase, to catalyze reactions from CO_2_ to CO and from CO_2_ to a methyl group, to which then acetyl-CoA is generated (Ragsdale and Pierce, 2008). High expression of genes encoding heterodisulfide reductase (*hdr*ABC2) were observed in the smooth mats. This enzyme is required for the final reaction steps of methanogenic pathways and is found in most methanogens (i.e., Euryarchaeota). Interestingly, the primary gene for methane production, *mcr*A, encoding methyl-coenzyme M reductase, was absent in all of the metatranscriptomes. This has previously been observed in metagenomes from smooth mats from Shark Bay and has been attributed to the presence of other potentially novel genes responsible for methanogenesis (Wong et al., 2018). Furthermore, both mat types exhibited an abundance of genes transcribed by microorganisms capable of surviving on methane which included methylotrophic pathways such as serine and ribulose monophosphate pathways. These pathways have been characterized in Alpha-, Beta-, and Gammaproteobacteria, Actinobacteria, Firmicutes, and Verrucomicrobia, and were actively abundant in both smooth and pustular mats (Chistoserdova, 2015). The abundance of active methane producing and consuming bacteria suggests the presence of symbiotic relationships and complete cycling of methane within Shark Bay microbial mats.

### Sulfur Cycling

Genes involved in sulfur metabolism were also found to be abundantly transcribed within smooth mat metatranscriptomes, with the most abundant gene transcripts being associated with the dissimilatory and assimilatory sulfate reduction pathways. Previous studies have shown higher rates of sulfate reduction in smooth mats when compared to pustular mats from Shark Bay using the silver foil technique (Pagès et al., 2014; Wong et al., 2015, 2018). The dissimilatory pathway is typically found in anaerobic bacterial and archaeal lineages (e.g., Deltaproteobacteria and Euryarchaeota), whereas the assimilatory pathway is found in a wider range of organisms (Pereira et al., 2011). It was recently reported that the majority of draft genomes constructed from Nilemah smooth mats encode partial and complete assimilatory sulfate-reduction pathways, indicating the potential importance of sulfur respiration in the Shark Bay ecosystems (Wong et al., 2018). In this study the elevated transcription of sulfur reduction pathways suggests that this is true for smooth mats, however, sulfur respiration does not appear to be a key active function in pustular mats investigated in our study. Additionally, genes encoding sulfide:quinone reductase (*sqr*) and sulfhydrogenase subunit gamma (*hyd*G) were also abundantly transcribed in smooth mats, suggesting that sulfide is being converted to polysulfide and subsequently assimilated as has been reported for intertidal microbial mats and in cultures of purple sulfur bacteria (Visscher et al., 1990; Visscher and van Gemerden, 1993).

### Nitrogen Cycling

There was no clear distribution of genes that encode nitrogen metabolizing pathways between both mat types. However, genes involved in nitrogen fixation were abundantly transcribed as well as other reduction pathways such as assimilatory and dissimilatory nitrate reduction. Recent taxonomic studies of Shark Bay microbial mats suggest that there are few nitrifiers present, leading to a potential incomplete cycling of nitrogen (Wong et al., 2015, 2018; Ruvindy et al., 2016). Any build-up of ammonium produced by nitrate reducing microorganisms is hypothesized to be assimilated by other members of the mat community (Wong et al., 2018). The elevated expression of glutamine synthase and glutamate synthase genes throughout the metatranscriptomes corroborates this. Additionally, a gene encoding methane/ammonia monooxygenase subunit A (*pmo*A-*amo*A) was transcribed in pustular mat metatranscriptomes. Genes encoding methane monooxygenase and ammonia monooxygenase share high sequence identity and therefore can represent members in phylogenetic groups of both methanotrophs (Alpha- and Gamma-proteobacteria) and ammonia-oxidizing nitrifying bacteria (Beta- and Gamma-proteobacteria) (Holmes et al., 2006). Therefore, due to the significant abundance of active Alphaproteobacteria in pustular mats, as well as there being incomplete cycling of nitrogen suggests that the *pmo*A-*amo*A gene is likely encoding methane monooxygenase.

### Functional Differences Between Mat Types, Diel Cycles, and Sampling Periods

The overall expression of individual functional genes indicated that there were no significant differences in day and night mat samples, however the results showed that samples grouped within their mat type and sampling period. This was also observed with the active microbial community distributions, which indicates that physiochemical conditions that shape microbial community structures as well as seasonal variations are significant factors influencing functional gene expression in these ecosystems. However, the combination of functional genes into the respective pathways indicated that PSI and PSII, as well as the Calvin–Benson cycle were transcribed more during the day time when peak oxygen production is known to occur (Louyakis et al., 2018). A potential reason for not observing diel changes in individual functional genes might be due to their stability. PSII is made up of two core subunits D1 and D2 encoded by *psb*A and *psb*D, while the two core subunits of PSI (A1 and A2) are encoded by *psa*A and *psa*B. Genes, *psa*A and *psa*B (PSI), and *psb*A (PSII) were highly transcribed in all the samples but indicated no significant diel variation. At night, PSII transcripts, such as *psb*A, have been shown to be more stable with a half-life of 7 h and play a role in regulating D1 production in some diazotrophic coccoid cyanobacteria, as well as being transcribed at night to form non-functional D1 proteins thereby preventing oxygen evolution during the nitrogen fixation period (Wegener et al., 2015). Additionally, cultured cyanobacteria have indicated that under high light intensity almost all of the PSI genes are downregulated to lower the susceptibility of the cells to damage, giving the appearance of no diel rhythm (Hihara et al., 1998). Furthermore, the lack of day/night differences can be attributed to the fact that only the upper few mm of phototrophic microbial mats are affected by day-night rhythms; lower parts of microbial mats typically contain microorganisms that are mainly surviving on leftover (more recalcitrant) organic matter; this part of the mat is still metabolically quite active, as previous sulfate reduction mapping efforts have shown (e.g., Wong et al., 2015). The homogenized sampling of the top ∼30 mm in this study may have decreased the overall observable transcription of functional genes that are directly affected by diel changes.

## Conclusion and Outlook

Extant microbial mats occurring in enhanced salinity environments are unique ecological niches representative of early life on Earth. Therefore, examining the underlying ecological drivers in active microbial mat communities is essential for gaining a better understanding of ancient ecosystems. The comparative analysis of smooth and pustular mat metatranscriptomes in this study provided a new outlook on how these communities respond and adapt to an elevated salinity and nutrient depleted environment, as well as either corroborating or disproving previous observations that were based on other previous genomic approaches. Discrepancies in the results and lack of observable diel changes were likely caused by sampling methodology, insufficient removal of rRNA and moderate sequencing depth. Therefore, future metatranscriptomic studies of Shark Bay microbial mats should try to laterally profile the mats (e.g., separate the upper phototrophic and lower heterotrophic regions), add an additional rRNA depletion step, and increase sequencing depth (e.g., minimum of 20 million reads per sample). Furthermore, future work should aim to employ an integrated metatranscriptomic and metagenomic approach to hypersaline mats to further reveal how specific groups microbes respond and adapt to different physiochemical conditions. The combination of these methods can concurrently investigate the transcriptional patterns of specific microbes, revealing their overall functional and metabolic activities within the whole community.

## Data Availability Statement

All codes, and scripts and expression/taxonomy data can be found on github.com/MACampbell91. Both pre-assembled and assembled reads have been deposited on osf.io/e94yg under the project “Shark Bay Transcriptomics”.

## Author Contributions

MAC, KG, PV, TM, and MJC conducted the field sampling of microbial mats. MAC and MJC done the laboratory sample processing. MAC, RW, and HW performed the statistical and computational analysis. All authors reviewed and edited the manuscript.

## Conflict of Interest

RW was employed by the company RAW Molecular Systems (RMS) LLC. The remaining authors declare that the research was conducted in the absence of any commercial or financial relationships that could be construed as a potential conflict of interest.
