# Supplementary material for: Functional Gene Expression in Shark Bay Hypersaline Microbial Mats: Adaptive Responses
Source: Front Microbiol. 2020 Nov 16;11:560336. doi: 10.3389/fmicb.2020.560336 (PMC7702295; doi:10.3389/fmicb.2020.560336)
Supplement: Supplementary file 1 [file Data_Sheet_1.docx]

Supplementary Information for

**Functional gene expression in Shark Bay hypersaline microbial mats: Adaptive Responses**

Matthew Campbell, Kliti Grice, Pieter T. Visscher, Therese Morris, Hon Lun Wong, Richard Allen White III, Brendan Burns, Marco JL Coolen

Matthew Campbell

Email: [macamp4591@gmail.com](mailto:macamp4591@gmail.com)

**This file includes:**

Figures S1 to S7

Tables S1 to S2

Supplementary Materials and Methods: provides further details of the sampling and site description, anddata analyses.


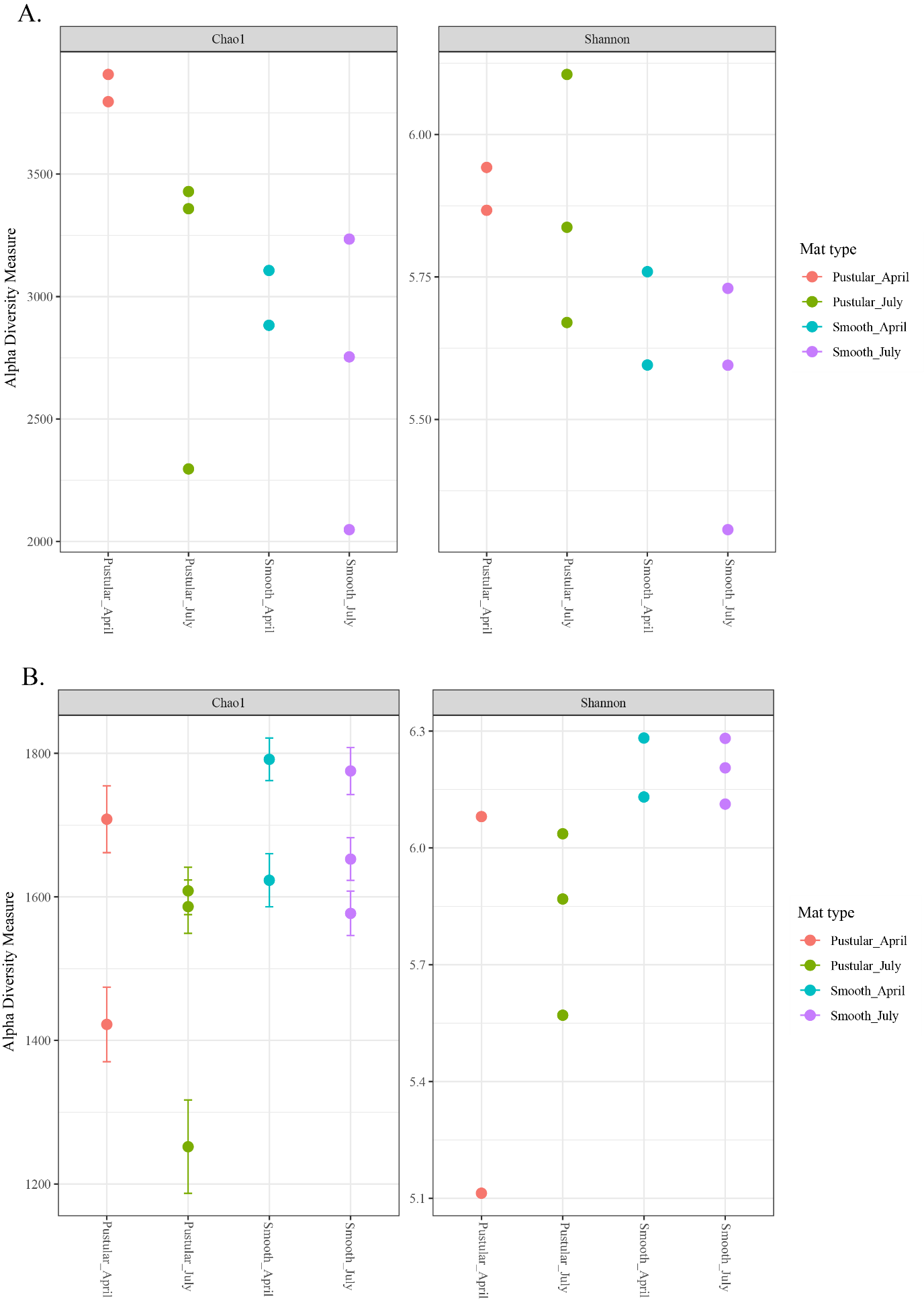


***Figure S1.*** Pustular and smooth mat alpha diversity represented by the Choa1 and Shannon’s diversity indices. A. SSU rRNA (SILVA) and B. Transcripts (RefSeq).

**
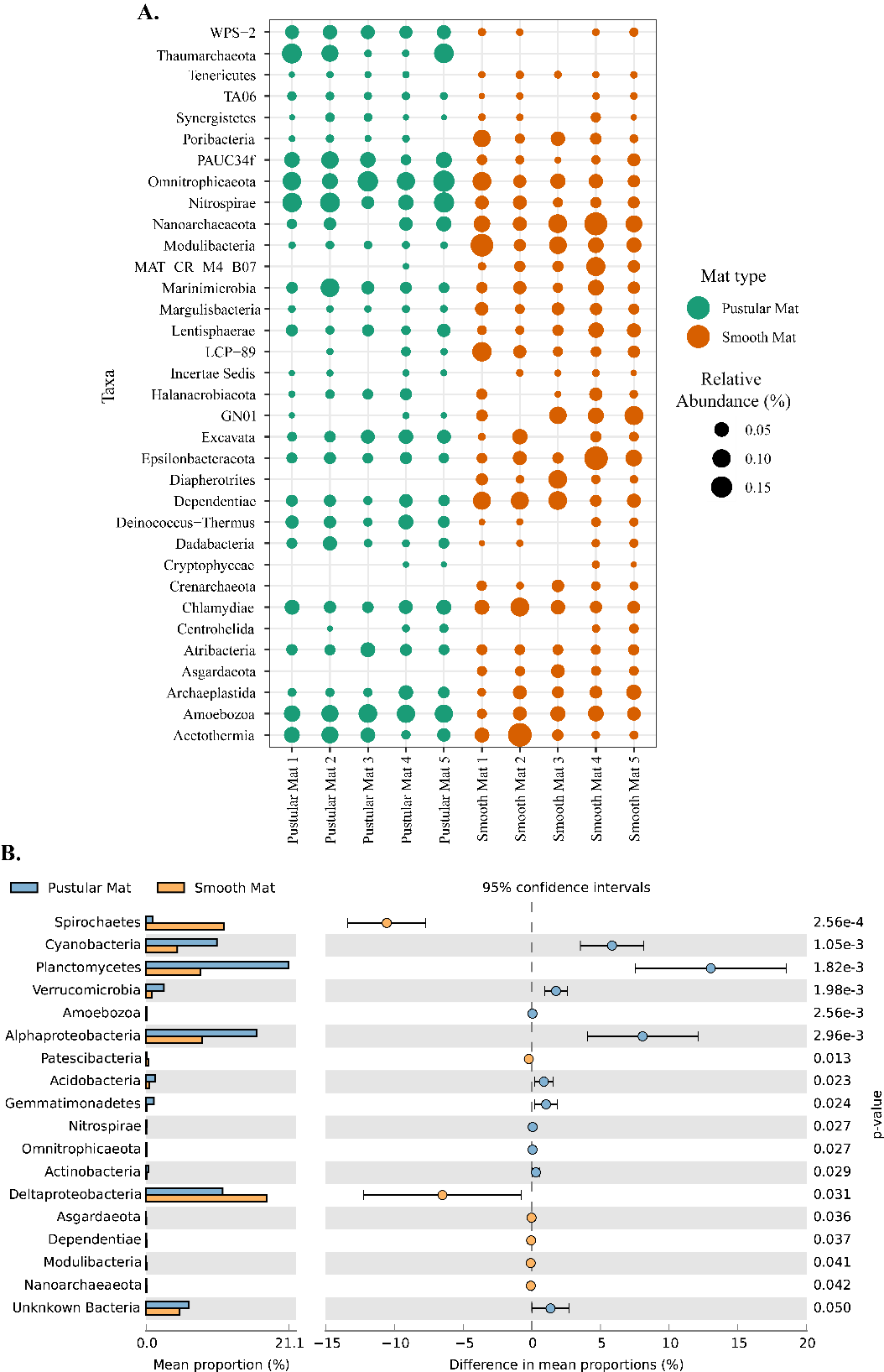
**

***Figure S2.*** Composition of Archaea, Bacteria and Eukaryote taxa in Nilemah smooth and pustular mats based on SSU rRNA genes (SILVA Database). A. Dot plot displaying the composition and abundance of the low abundant taxa. B. Extended error bar plot identifying significant differences (p < 0.5) between mean proportions of microbial taxa in smooth and pustular mats (ANOVA, Tukey's t‐test). Mats 1, 2 and 3 were sample in July 2016; mats 4 and 5 were sampled in April 2017. Pustular and smooth mats 2 and 3 are the paired samples from July 2016.


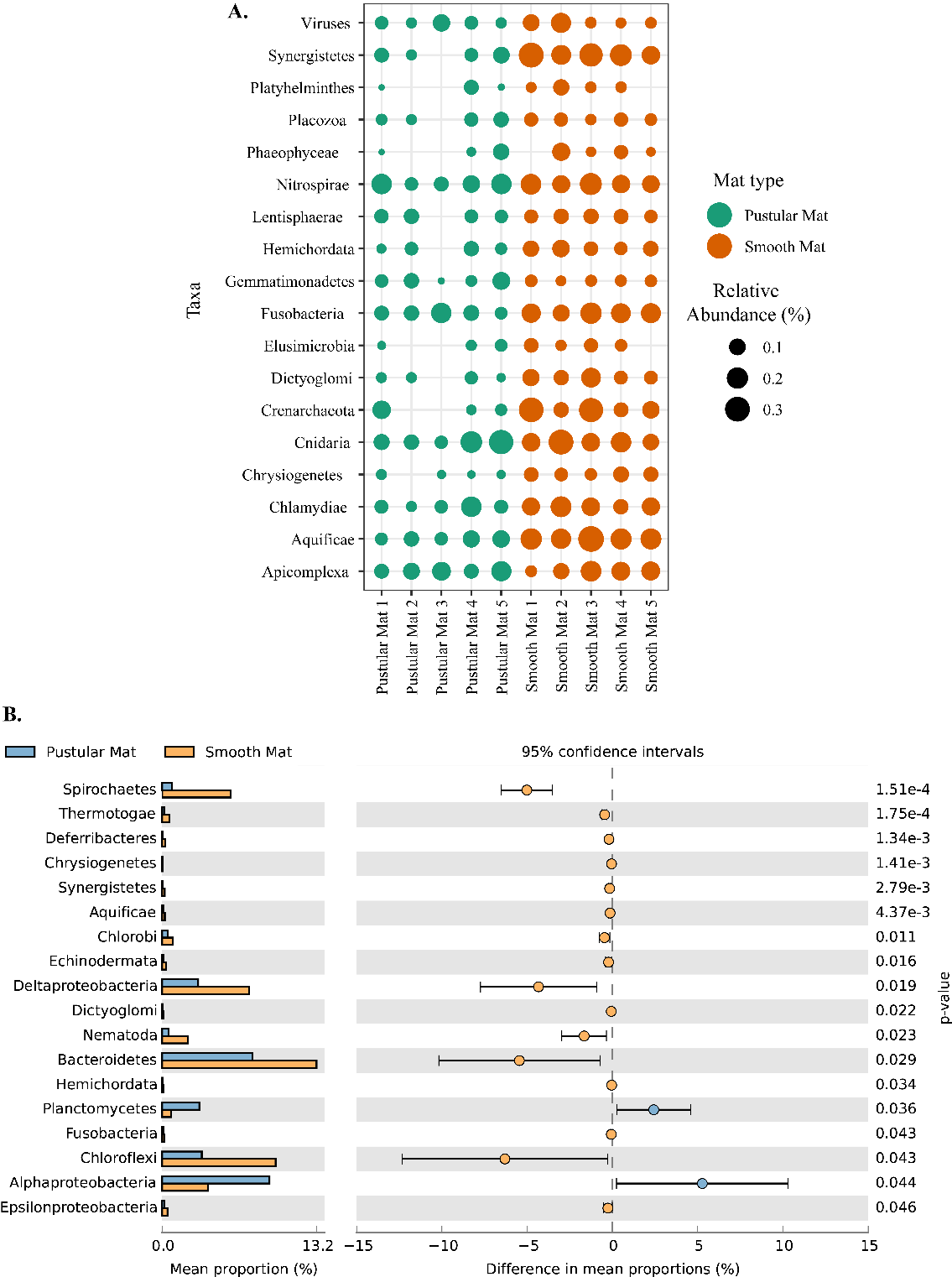


***Figure S3.*** Composition of Archaea, Bacteria and Eukaryote taxa in Nilemah smooth and pustular mats based on transcripts (RefSeq Database). A. Dot plot displaying the composition and abundance of the low abundant taxa. B. Extended error bar plot identifying significant differences (p < 0.5) between mean proportions of microbial taxa in smooth and pustular mats (ANOVA, Tukey's t‐test). Mats 1, 2 and 3 were sample in July 2016; mats 4 and 5 were sampled in April 2017. Pustular and smooth mats 2 and 3 are the paired samples from July 2016.


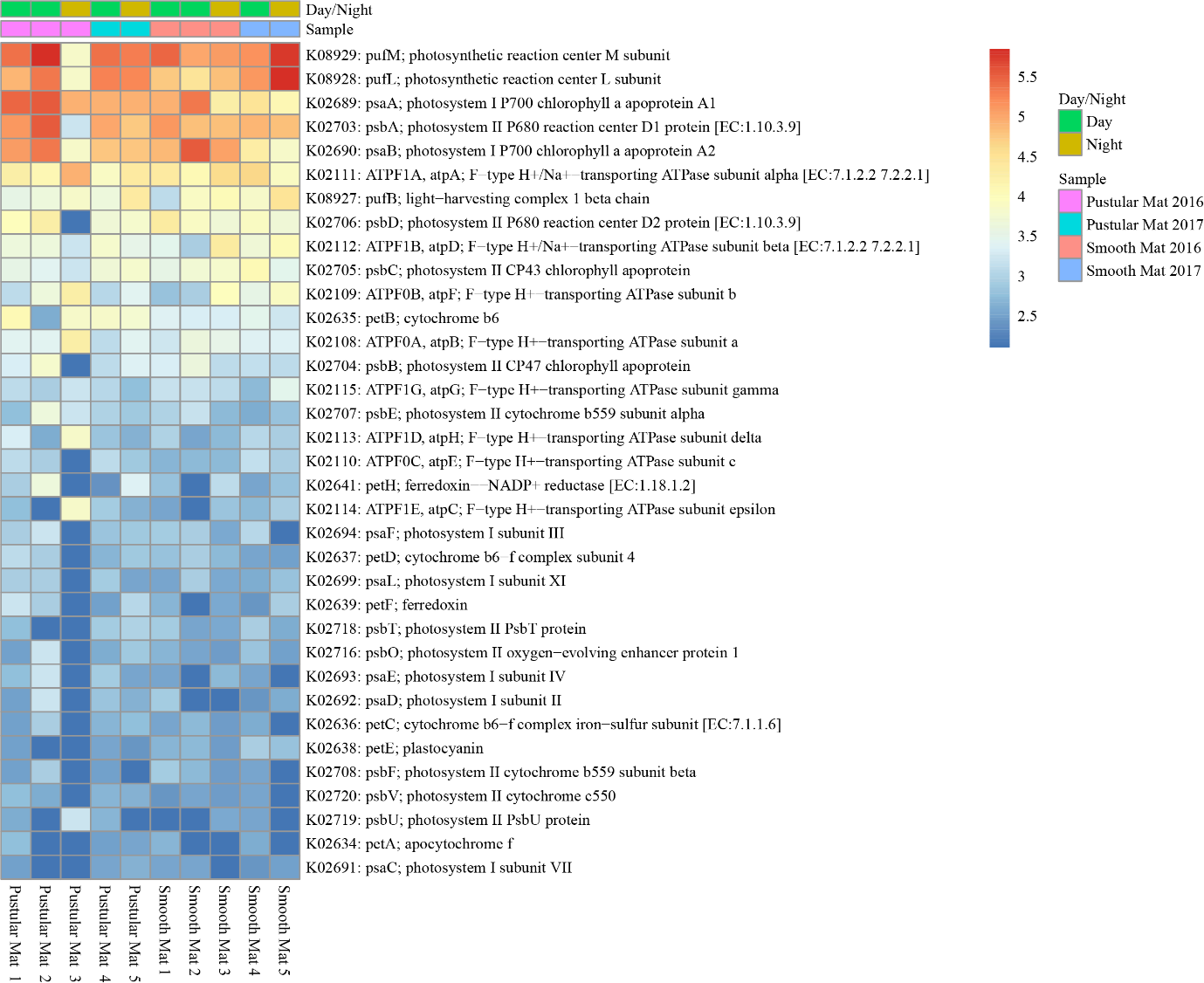


***Figure S4.***Heatmap showing 35 transcribed genes (>10) in Nilemah smooth and pustular mat metatranscriptomes related to photosynthesis. Differential analysis of the transcribed genes was calculated from the variance stabilising transformation of KO count data using the DESeq2 package in R. A gradient from red to blue indicates gene abundance across samples with red representing genes that are highly transcribed and blue indicating genes that have lower relative transcription. Pustular and smooth mats 2 and 3 are the paired samples from July 2016.


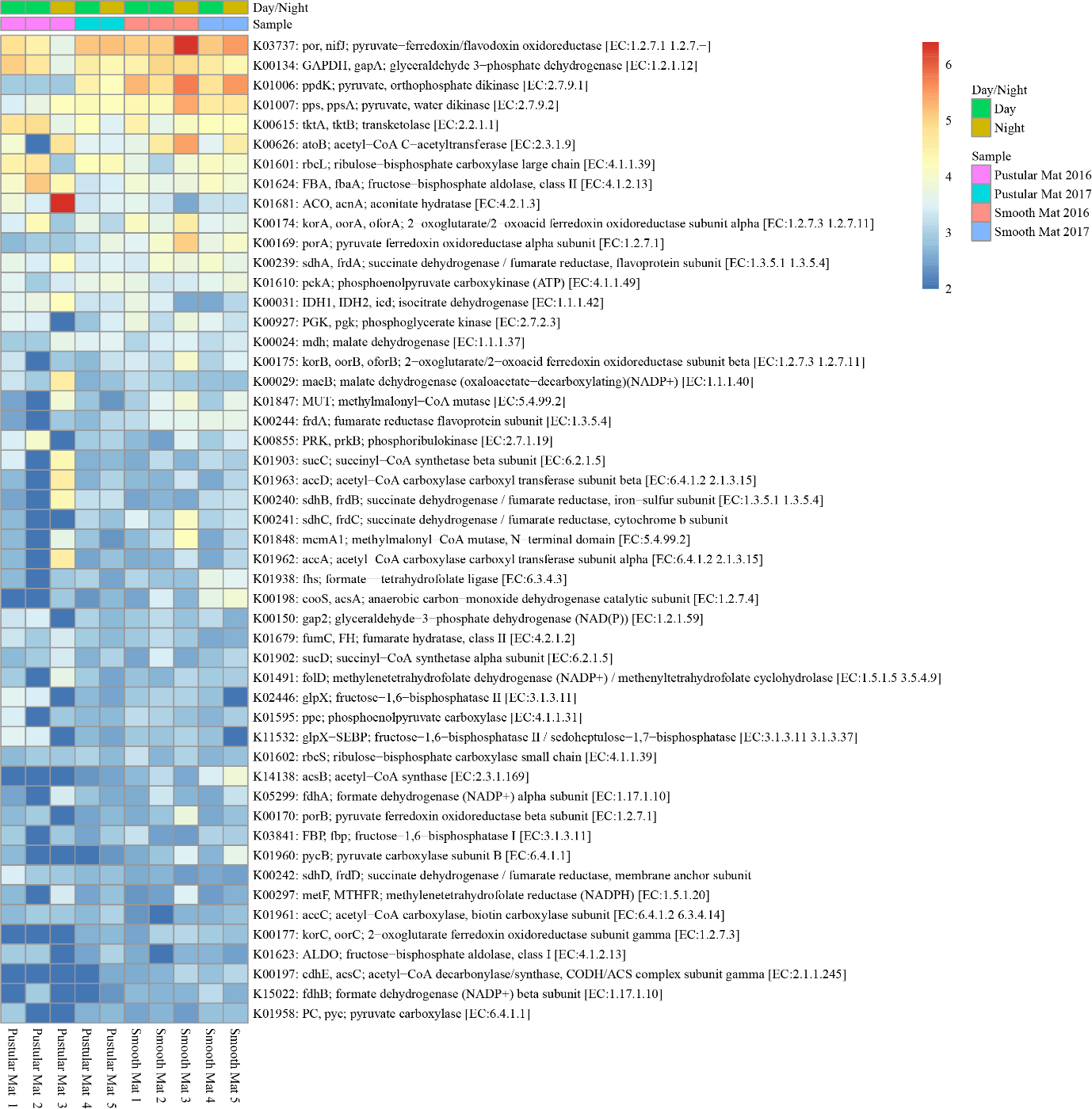


***Figure S5.***Heatmap showing the top 50 out of 67 transcribed genes (>10) in Nilemah smooth and pustular mat metatranscriptomes related to carbon fixation. Differential analysis of the transcribed genes was calculated from the variance stabilising transformation of KO count data using the DESeq2 package in R. A gradient from red to blue indicates gene abundance across samples with red representing genes that are highly transcribed and blue indicating genes that have lower relative transcription. Pustular and smooth mats 2 and 3 are the paired samples from July 2016.


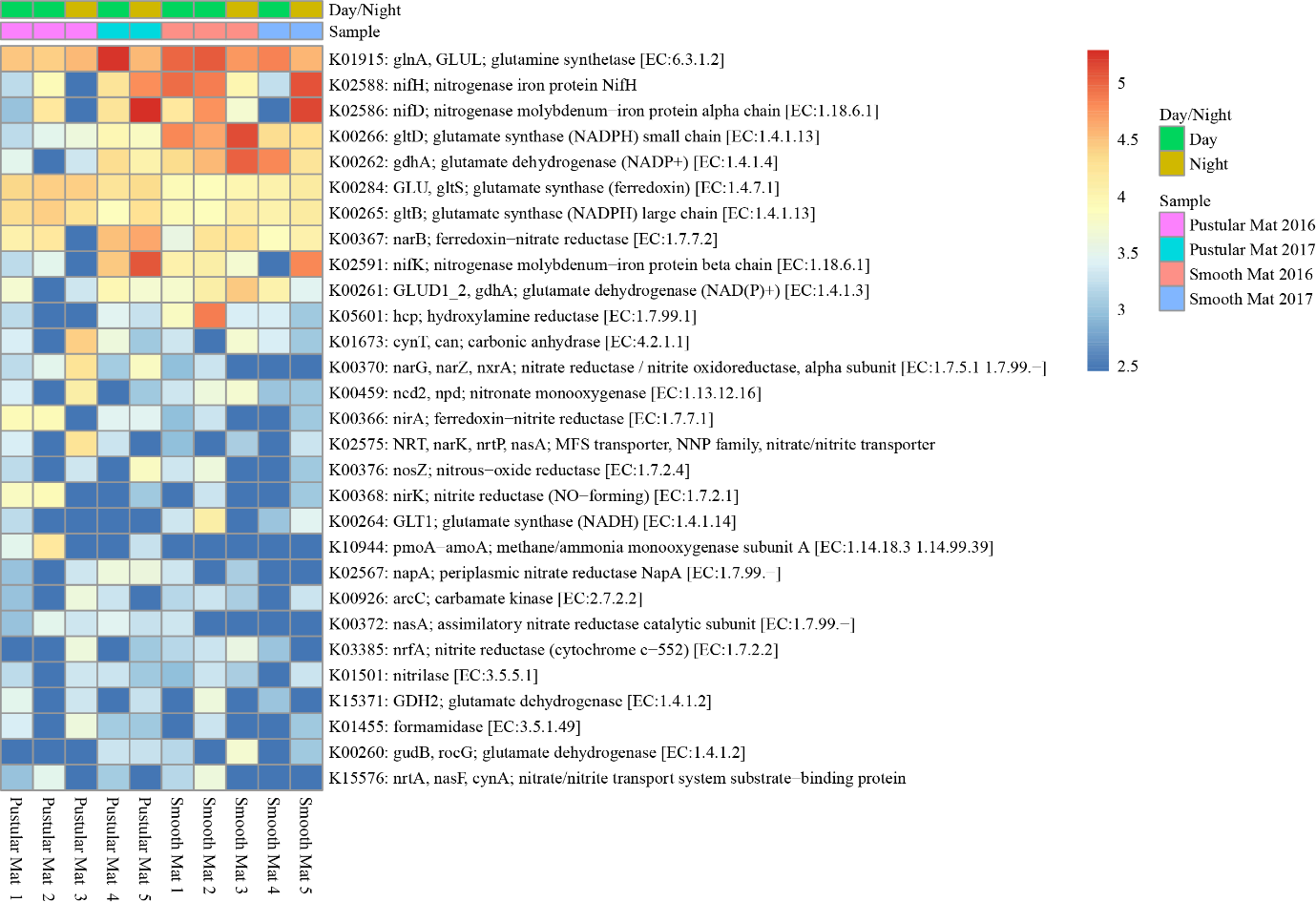


***Figure S6.*** Heatmap showing 29 transcribed genes (>10) in Nilemah smooth and pustular mat metatranscriptomes related to nitrogen metabolism. Differential analysis of the transcribed genes was calculated from the variance stabilising transformation of KO count data using the DESeq2 package in R. A gradient from red to blue indicates gene abundance across samples with red representing genes that are highly transcribed and blue indicating genes that have lower relative transcription. Pustular and smooth mats 2 and 3 are the paired samples from July 2016.


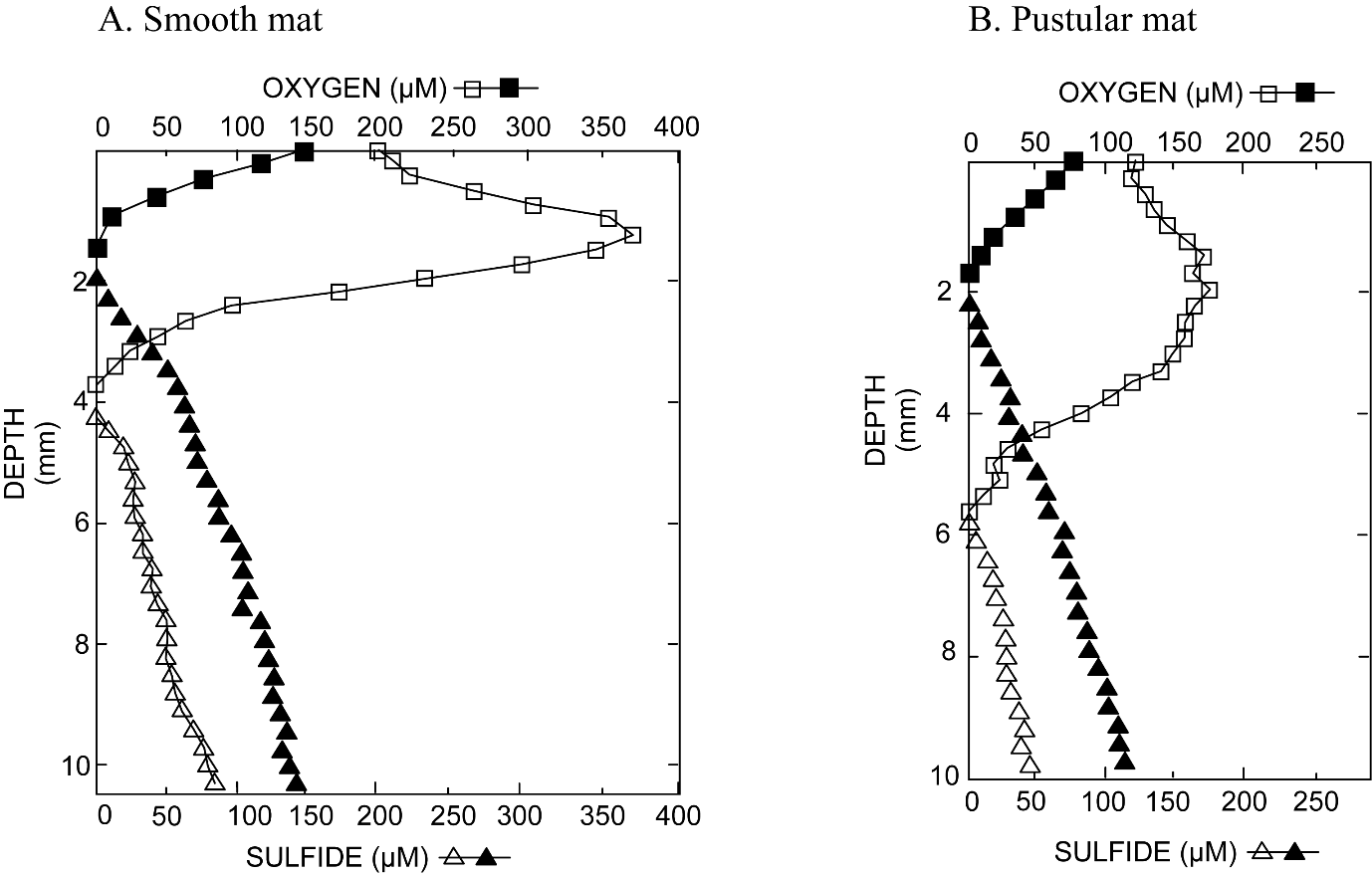


**Figure S7.***In situ* depth profiles of oxygen and sulfide concentrations in Shark Bay microbial mats (A. smooth mats and B. Pustular mat) measured with needle electrodes during the 2016 field trip. Oxygen and sulfide concentrations were measured during peak photosynthesis between noon and 2:00 pm and the end of the night, between 3:00 and 5:00 am. Squares represent oxygen concentrations, triangles represent sulfide concentrations. Methods detailed in Wong et al., 2015).

***Table S1***Summary of the mat types collected with sampling dates and times, and field measurements of salinity, pH and water temperature. *Indicating paired Day/Night samples in the 2016 sampling trip. Elevation as measured by DGPS, approx. values derived from contoured LiDAR and hyperspectral bathymetry DTM’s (see Morris et al in press for method(Morris et al., 2020)). Biogeomorphic Unit, TMMU = Transitional Microbilite Mosaic Unit, MPU = Mat Platform Unit (terminology after Morris et al in press). Geomorphic unit, terminology after Nordstrom and Jackson, 2012(Nordstrom and Jackson, 2012). Note: Samples were collected under spring tide conditions.

| **Sample ID** | **Sampling date** | **UTM Coordinates (Zone 50)** | **Day/Night** | **Sampling time** | **Subsampling time** | **Salinity (‰)** | **pH** | **Water temp (°C)** | **Elevation (mAHD)** | **Biogeomorphic unit** | **Geomorphic setting** | **Morphological feature** |
| --- | --- | --- | --- | --- | --- | --- | --- | --- | --- | --- | --- | --- |
| Pustular Mat 1 | 7/5/2016 | 210428, 7071152 | Day | 12:40 PM | 3:10 PM | 67 | 7.94 | 13.2 | -0.32 | TMMU | inter-tidal terrace front | microbialite ridge crest |
| Pustular Mat 2* | 7/5/2016 | 210386, 7071116 | Day | 12:10 PM | 3:00 PM | 66 | 8.06 | 13.2 | 0.01 | MPU | mid-intertidal terrace | continuous mat sheet |
| Pustular Mat 3* | 7/6/2016 | 210386, 7071116 | Night | 11:45 PM | 5:00 AM | 66 | 8.28 | 13.1 | 0.01 | MPU | mid-intertidal terrace | continuous mat sheet |
| Pustular Mat 4 | 4/10/2017 | 210421, 7071167 | Day | 8:30 AM | 11:45 PM | 70 | 7.51 | 18 | ~ -0.25 | TMMU | inter-tidal terrace front | microbialite ridge crest |
| Pustular Mat 5 | 4/11/2017 | 210421, 7071167 | Night | 10:00 PM | 4:15 AM | 70 | 8.12 | 16 | ~ -0.25 | TMMU | inter-tidal terrace front | microbialite ridge crest |
| Smooth Mat 1 | 7/5/2016 | 210390, 7071195 | Day | 12:00 PM | 2:45 PM | 66 | 7.99 | 13.2 | -0.42 | TMMU | inter-tidal terrace front | swale between microbialite ridges |
| Smooth Mat 2* | 7/5/2016 | 210387, 7071191 | Day | 12:30 PM | 2:30 PM | 67 | 7.93 | 13.2 | -0.42 | TMMU | inter-tidal terrace front | swale between microbialite ridges |
| Smooth Mat 3* | 7/6/2016 | 210387, 7071191 | Night | 12:00 AM | 5:10 AM | 66 | 8.28 | 13.1 | -0.42 | TMMU | inter-tidal terrace front | swale between microbialite ridges |
| Smooth Mat 4 | 4/10/2017 | 210421, 7071167 | Day | 8:45 PM | 12:00 PM | 70 | 7.51 | 18 | ~ -0.4 | TMMU | inter-tidal terrace front | swale between microbialite ridges |
| Smooth Mat 5 | 4/11/2017 | 210421, 7071167 | Night | 10:15 PM | 4:20 PM | 70 | 8.12 | 16 | ~ -0.4 | TMMU | inter-tidal terrace front | swale between microbialite ridges |

***Table S2***Illumina HiSeq 2500 pair-end sequencing read output with percentage summaries of trimmed and aligned sequences, number of assembled transcripts and number of annotations for Phyloflash, DIAMOND and eggnog-mapper.

|  |  | Percentage of trimmed and aligned sequences | | | |  | # annotations | | |
| --- | --- | --- | --- | --- | --- | --- | --- | --- | --- |
| Sample Name | Paired Reads | Trimmed  reads | Remaining reads | aligned 0 times | aligned >1 times | # transcripts assembled | PhyloFlash (SSU rRNA SILVA) | DIAMOND (NCBI RefSeq) | eggNOG-mapper (KOs) |
| Pustular Mat 1 | 10737329 | 8.9 | 91.1 | 7.1 | 92.9 | 26890 | 1474649 | 15713 | 10275 |
| Pustular Mat 2 | 9066195 | 7.7 | 92.3 | 7.6 | 92.4 | 9393 | 1652757 | 3842 | 2670 |
| Pustular Mat 3 | 8869056 | 11.8 | 88.2 | 63.1 | 36.9 | 29879 | 820936 | 18363 | 7119 |
| Pustular Mat 4 | 9184291 | 9.9 | 90.1 | 9.4 | 90.6 | 29423 | 1827324 | 14175 | 13866 |
| Pustular Mat 5 | 9938032 | 8.6 | 91.4 | 8.6 | 91.4 | 29383 | 1690603 | 14000 | 13261 |
| Smooth Mat 1 | 9621836 | 8.5 | 91.5 | 8.4 | 91.6 | 25348 | 1707460 | 11845 | 10096 |
| Smooth Mat 2 | 9771353 | 9.4 | 90.6 | 15.6 | 84.4 | 63494 | 905558 | 20570 | 18172 |
| Smooth Mat 3 | 9153948 | 9.4 | 90.6 | 16.8 | 83.2 | 57592 | 755780 | 19647 | 22330 |
| Smooth Mat 4 | 9214600 | 8.7 | 91.3 | 9.5 | 90.5 | 24822 | 1293097 | 22851 | 12490 |
| Smooth Mat 5 | 9956952 | 8.5 | 91.6 | 9.7 | 90.3 | 26087 | 1703240 | 12504 | 12427 |

**Supplementary Materials and Methods**

**Sampling and site description**

Microbial mats were sampled from the Nilemah tidal flat (26°27'02.9"S 114°05'39.3"E) located in the southern area of Hamelin Pool, Shark Bay, Western Australia (Fig. 1).Restricted circulation in conjunction with high rates of evaporation and limited rainfall leads to the water in Hamelin Pool to be hypersaline (60 to 70 ppt) with pH ranges between 7.5 and 8(Jahnert and Collins, 2013; Suosaari et al., 2016).The Nilemah tidal flat is shallow and characterized by a littoral gradient that varies from 20 to 150 cm km^−1^ restricting tidal influxes and causing laterally well‐defined tidal zonation (Jahnert and Collins, 2013). Fluctuations in the water level within the intertidal and upper subtidal zone occur on both semidiurnal (astronomical tide) frequency but also at seasonal periodicity with highest water in March/April and lowest water levels in October/November. Recent studies of water elevation within Hamelin Pool found that up to 60% of the observed variation in surface elevation was attributable to meteorological drivers (Burne and Johnson, 2012; Suosaari et al., 2016). In addition, wind induced set down isparticularly important in the Nilemah tidal flat since it can prevent the inundation of the mat platform by the tide for extended periods, however this phenomenon is most likely to affect the setting during windy months and not during the time of sampling in this study (April and July)(Morris et al., 2020).

Smooth and pustular microbial mats were sampled from theintertidal and uppermost subtidal zone of the Nilemah tidal flat (Fig. 2). These mat types were chosen for this study since they are the most common mat types for this area and parallel metagenomics datasets are available for comparison. Smooth mats are uniformly laminated with a pale brown-green surface overlaying a light to dark green second layer followed by a third purple layer and fourth black layer and were located in small depressions between mat covered microbialite ridges that remained submerged at low tide. In contrast, pustular mats are dark brown at the surface with jelly‐like pustules composed of green, gold and purple mucilage. This mat type was located at on the crests of microbialite ridges between depressions in the shallow subtidal zone and as continuous sheets across the intertidal platform where it was exposed at low tide.Microbial mats were sampled in the field using aluminium push cores (20 cm long x 10 cm wide) for organic geochemical and genomic analyses during day and night at low tide. The push cores were wrapped in aluminium foil (annealed at 550°C) prior to sampling.

**References for Supplementary Information**

Burne, R. V., Johnson, K., 2012. Sea-level variation and the zonation of microbialites in Hamelin Pool, Shark Bay, Western Australia. Mar. Freshw. Res. 63, 994–1004.

Jahnert, R.J., Collins, L.B., 2013. Controls on microbial activity and tidal flat evolution in Shark Bay, Western Australia. Sedimentology 60, 1071–1099.

Morris, T.E., Visscher, P.T., O’Leary, M.J., Fearns, P.R.C.S., Collins, L.B., 2020. The biogeomorphology of Shark Bay’s microbialite coasts. Earth-Science Rev.

Nordstrom, K.F., Jackson, N.L., 2012. Physical processes and landforms on beaches in short fetch environments in estuaries, small lakes and reservoirs: A review. Earth-Science Rev.

Suosaari, E.P., Reid, R.P.R., Araujo, T.A.A., Playford, P.E., Holley, D.K., McNamara, K.J., Eberli, G.P., 2016. Environmental Pressures Influencing Living Stromatolites in Hamelin Pool, Shark Bay, Western Australia. Palaios 31, 483–496.

Wong, H.L., Smith, D.L., Visscher, P.T., Burns, B.P., 2015. Niche differentiation of bacterial communities at a millimeter scale in Shark Bay microbial mats. Sci. Rep. 5, 15607.
